# SpatialCPie: an R/Bioconductor package for spatial transcriptomics cluster evaluation

**DOI:** 10.1186/s12859-020-3489-7

**Published:** 2020-04-29

**Authors:** Joseph Bergenstråhle, Ludvig Bergenstråhle, Joakim Lundeberg

**Affiliations:** 1 Science for Life Laboratory, KTH Royal Institute of Technology, Stockholm, Sweden; 2 Department of Bioengineering, Stanford University, California, USA

**Keywords:** Spatial transcriptomics, Cluster analysis, Data visualization, R package

## Abstract

**Background:**

Technological developments in the emerging field of spatial transcriptomics have opened up an unexplored landscape where transcript information is put in a spatial context. Clustering commonly constitutes a central component in analyzing this type of data. However, deciding on the number of clusters to use and interpreting their relationships can be difficult.

**Results:**

We introduce SpatialCPie, an R package designed to facilitate cluster evaluation for spatial transcriptomics data. SpatialCPie clusters the data at multiple resolutions. The results are visualized with pie charts that indicate the similarity between spatial regions and clusters and a cluster graph that shows the relationships between clusters at different resolutions. We demonstrate SpatialCPie on several publicly available datasets.

**Conclusions:**

SpatialCPie provides intuitive visualizations of cluster relationships when dealing with Spatial Transcriptomics data.

## Background

Clustering is a standard analysis operation used for grouping entities in complex datasets to bring order and find patterns of similarity. Typically, clusters are used for identification purposes and further downstream analysis, e.g., statistical identification of key drivers of dissimilarity. The clustering can be conducted in various ways. Common techniques include k-means clustering, hierarchical clustering, DBSCAN, or MCL [1]. Most clustering methods require prespecifying the number of clusters to use or otherwise choosing suitable hyperparameters for the dataset at hand.

Spatial Transcriptomics (ST) is a recent method to obtain spatial information during RNA-seq experiments [2]. Briefly, barcoded capture probes are grouped into “spots” and printed on a glass array. The tissue section is placed on the array and permeabilized so that transcripts diffuse down to the capture probes. After sequencing, the barcodes of the probes can be used to map the transcripts back to the spot in which they were captured.

A common step in analyzing ST data is to cluster the gene expression profiles of the spots in order to identify and annotate regions of interest in the tissue section. This could, for example, be used to identify tumor regions or discover intra-tumor heterogeneity hidden to the human eye [3]. However, selecting appropriate hyperparameters, e.g., the right number of clusters to use, poses a challenge in these types of analyses. Indeed, it is often necessary to try out different sets of hyperparameters, as each may provide distinct insights about the data. Moreover, the relationships between clusters are not always clear, and common visualizations strategies for high dimensional data, for example based on t-SNE, often produce results that are difficult to interpret [4]. An additional obstacle is the fact that each barcoded spot in ST normally captures multiple cells. Consequently, gene expression measurements are derived from mixtures of cells, obfuscating cluster-based cell-type identification.

While tools exist for visualizing clusters in the context of ST data, none fully address the above concerns. Most prominently, the ST viewer [5] can visualize clusters spatially but classifications are binary and only a limited number of clustering algorithms are supported.

Here, we present SpatialCPie, an easy-to-use R package that gives the user an intuitive understanding of how clusters in ST data are related to each other and to what extent each region on the two-dimensional ST array is associated with each cluster. SpatialCPie is designed to be used as part of an R workflow, giving the user a high degree of flexibility to customize and quickly iterate their analyses. The data is clustered at multiple *resolutions*—i.e., with different numbers of clusters or hyperparameter settings—thereby avoiding the need to prespecify a single set of hyperparameters for the analysis, and the user can freely define which clustering algorithm to use. The results are visualized in two ways: with a *cluster graph* [6] that shows how clusters overlap between different resolutions and with two-dimensional *array plots* in which each spot is represented by a pie chart indicating its similarity to the different cluster centroids.

Historically, pie charts have frequently been used to display spatial data on geographical maps [7, 8]. Recently, with the advent of spatial omics and in a similar vein as the work presented here, analogous visualizations have also successfully been applied to tissue maps [9].

## Implementation

The user interface of SpatialCPie is implemented in Shiny [10]. The interface consists of two main components: the cluster graph and the array plots, both described in detail below.

### Cluster graph

**Fig. 1:**
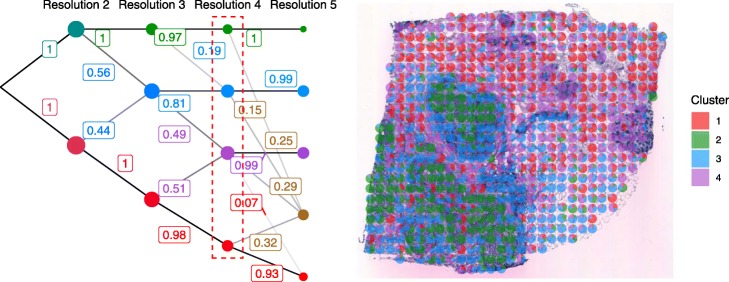
Left: The cluster graph. Edge opacity indicates the proportion of spots in the higher-resolution cluster that originate from the lower-resolution cluster. Right: Array plot. Pie charts show gene expression similarity between spatial regions and the cluster centroids. In both plots, expression profiles are projected into color space by PCA, so that similar clusters have similar colors

The cluster graph (Fig. 1, left) is a graph that visualizes the relationships between clusters over different resolutions. Clusters are represented as nodes in the graph, and edges show the degree to which clusters in consecutive resolutions overlap. Specifically, the opaqueness of an edge indicates the proportion of spots in the higher-resolution cluster that also exist in the lower-resolution cluster. The user can set a threshold on the proportion so that less informative edges—those representing only very small overlaps—are removed. Cluster relationships are further visualized by encoding the mean expression profile of each cluster in color space so that nodes constituting spots with similar expression have similar colors. The user can hover a node to see a summary of the most expressed genes in the cluster.

The cluster graph shows the ancestry of clusters and allows the user to reconcile insights from different cluster resolutions (“Human developmental heart” section).

### Array plot

The array plot (Fig. 1, right) is a graphical representation of the ST array. A pie chart for each spot shows the similarity score between the spot and the cluster centroids. The similarity score between spot *s* and cluster *k* is defined as

1 $$ \text{score}(s,k) = \exp\left(-\lambda\text{RMSD}\left(x_{s}, \text{mean}\left\{x_{s'}\right\}_{s'\in C(k)} \right) \right),   $$

where *x*_*i*_ is the gene expression vector of spot *i*,*C*(*k*) is the set of spots in cluster *k*,RMSD(*a*,*b*) is the root-mean-square deviation between gene vectors *a* and *b*, and *λ* is a user-selectable constant.

The pie charts relativize cluster assignments, making it possible to identify spatial trends in gene expression (fig. S2).

### Sub-clustering

In a typical analysis of ST data, it is often the case that some parts of the tissue cluster clearly at a low resolution and are of less interest for further exploration. Meanwhile, other regions may be interesting to study in finer detail by sub-clustering. This can be achieved by using the tool iteratively (“Human developmental heart” section and Fig. 3).

## Results

SpatialCPie can be used to analyze any dataset with spatially distributed count data. Here, we demonstrate its utility on three publicly available ST datasets [11–13]: the human developmental heart (“Human developmental heart” section), breast cancer in situ (section S2.1), and melanoma (section S2.2). In all cases, we normalize the data using Seurat [14] before passing it to SpatialCPie.

### Human developmental heart

**Fig. 2:**
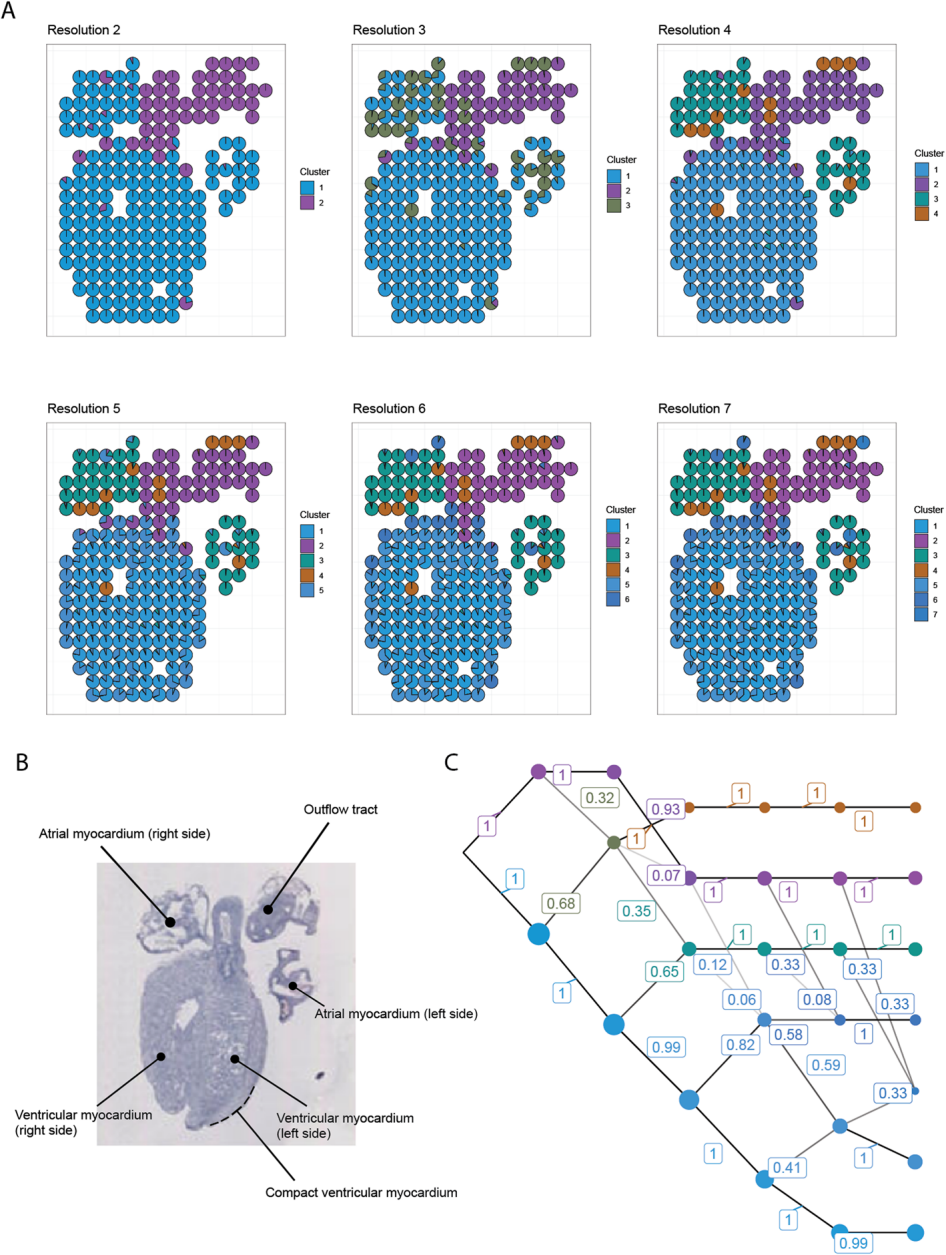
The human developmental heart. **a** Array plots. **b** H&E stain of the sample with annotated anatomical areas. **c** Cluster graph. The small color differences between the ventricular clusters (blue) indicate that their expression profiles are similar compared to the other clusters

The tissue section is taken from a 5-week-old heart with well-defined anatomical regions (Fig. 2b).

**Fig. 3:**
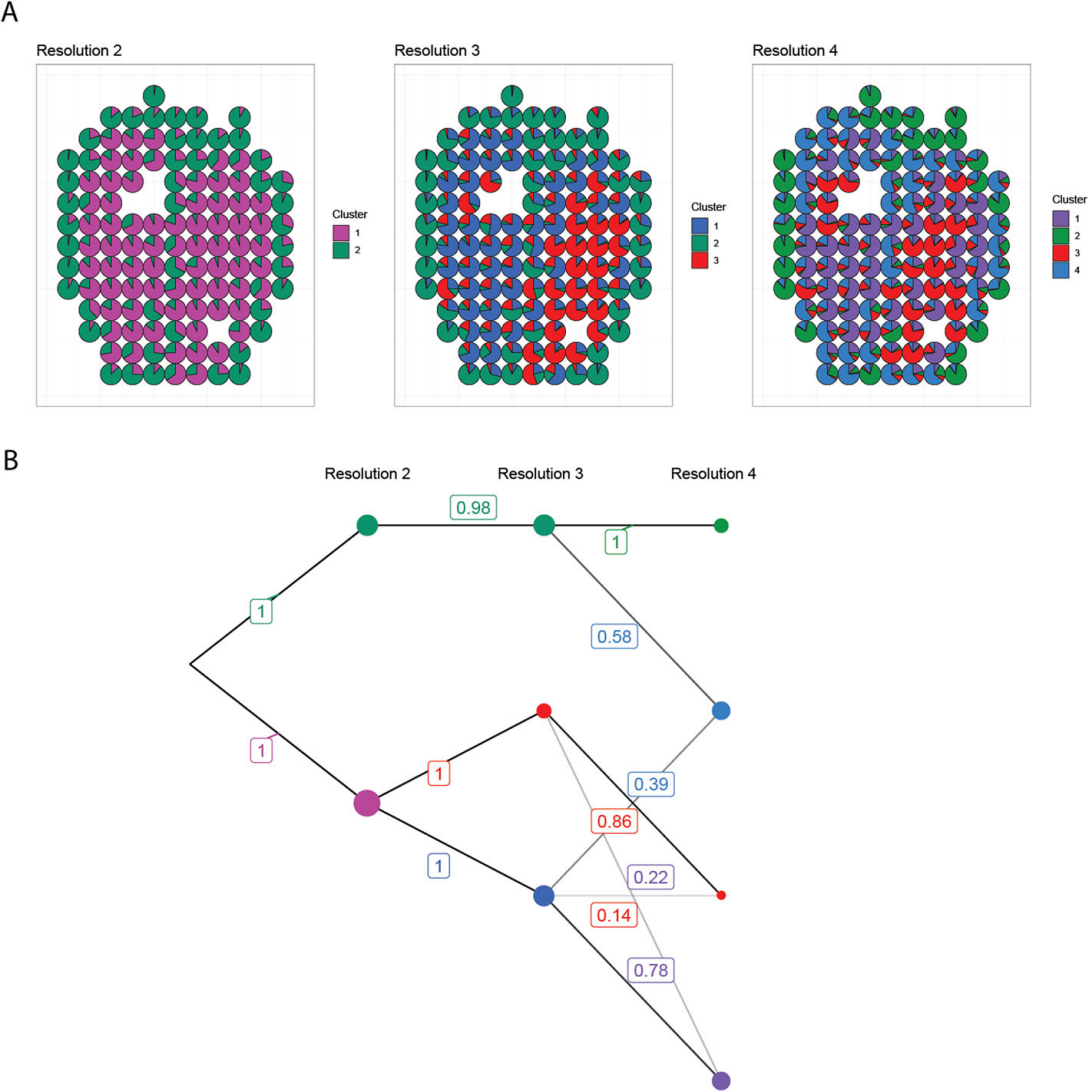
Sub-clustering of the left and right ventricle of the developmental heart. **a** Array plots. Resolution 2 shows a rim-like structure spanning the periphery of the tissue (compact ventricular myocardium). Resolution 3 shows evidence of gene expression differences between the left and right ventricle. Cluster 4 in resolution 4 indicates another subtle rim-like structure contained within the outermost rim. **b** Cluster graph. The left and right ventricles share ancestry, suggesting relatedness. The inner rim structure shares ancestry with the outer rim and one of the ventricles

The array plots (Fig. 2a) and cluster graph (Fig. 2c) show a clear separation between the outflow tract, atria, and ventricles across resolutions. It is also evident that the outflow tract is highly homogeneous; most of its spots exhibit high similarity scores to a single cluster (cluster 2), and this cluster is clearly separated in color space from other clusters.

There is evidence of subtle differences in gene expression within the ventricles, but the clusters there are more similar to each other than to other clusters, as indicated by their colors and shared ancestry (Fig. 2c). Sub-clustering the ventricles (Fig. 3) reveals the compact ventricular myocardium that spans the periphery of the tissue. Curiously, we also find that the left and right ventricle exhibit slightly different cluster affinities, suggesting that their differences could be an interesting property to investigate further.

## Conclusion

SpatialCPie provides a user-friendly interface for analyzing clusters in ST data and uses visualization techniques to help the analyst uncover and explore hidden gene expression patterns. Concretely, clustering is done at multiple resolutions, each providing a different level of granularity of the patterns in the data. Clusters over different resolutions are hierarchized in a cluster graph, and their spatial distributions are visualized in array plots. The array plots relativize cluster membership for each spatial region, thereby exposing gradients in gene expression that otherwise would be difficult to observe.

Overall, we find that the visual clues from looking at multiple cluster resolutions on the array plots, the relationships between the clusters in the cluster graph, as well as their color-coded expression profiles together give a comprehensive view of the spatial gene expression landscape in tissues.

## Availability and requirements

**Project name** SpatialCPie

**Project home page** https://github.com/jbergenstrahle/SpatialCPie

**Operating system(s)** Platform independent

**Programming language** R

**License** MIT

## Data Availability

The fetal heart dataset was obtained from the authors of [11].

## Abbreviations

ST: Spatial transcriptomics
